# Atg5-Mediated Lipophagy Induces Ferroptosis in Corneal Epithelial Cells in Dry Eye Disease

**DOI:** 10.1167/iovs.65.14.12

**Published:** 2024-12-05

**Authors:** Xin Zuo, Hao Zeng, Xue Yang, Dalian He, Bowen Wang, Jin Yuan

**Affiliations:** 1 State Key Laboratory of Ophthalmology, Zhongshan Ophthalmic Center, Sun Yat-Sen University, Guangdong Provincial Key Laboratory of Ophthalmology and Visual Science, Guangzhou, China

**Keywords:** dry eye disease, lipophagy, atg5, ferroptosis, lipid droplets

## Abstract

**Purpose:**

Ferroptosis occurred in corneal epithelial cells has been implicated in the inflammation in dry eye disease (DED). Given the proposed link between ferroptosis and autophagy, this study aims to investigate the role of autophagy in driving ferroptosis in corneal epithelial cell and enrich the pathogenesis underlying DED.

**Methods:**

DED models were established in C57BL/6 mice via scopolamine injection and in human corneal epithelial cell line (HCEC) using hyperosmotic medium. Lipidomic and transcriptomic analysis were conducted to assess lipid metabolism and regulatory pathways. Atg5 expression was manipulated in vivo using cholesterol-modified small interfering RNA. Lipid droplets (LDs) and lysosomes were labeled with BODIPY 493/503 and Lysotracker Red DND-99, respectively. Western blot, immunofluorescence (IF) staining, co-immunoprecipitation (CO-IP), transmission electron microscopy and microplate reader were used to explore protein expressions and interactions, cellular structures, and free fatty acid (FFA) content.

**Results:**

Our results revealed that autophagy was activated in DED, as evidenced by lipidomic and transcriptomic analyses. Enhanced lipophagy was observed in HCECs exposed to hyperosmolarity, manifested by lysosome-LD co-localization and autophagic vacuoles containing LDs. Upregulation of Atg5 promoted lipophagy, leading to elevated cellular FFA levels, lipid peroxidation, and expression of ferroptosis markers. Interaction between Atg5 and perilipin3 was confirmed through CO-IP and IF. In the DED mouse model, Atg5 inhibition effectively ameliorated corneal damage, suppressed ferroptosis and ocular surface inflammation.

**Conclusions:**

Our findings highlight the pivotal role of Atg5-mediated lipophagy in driving ferroptosis in corneal epithelial cells in DED, proposing Atg5 as a promising therapeutic target for mitigating ferroptosis-induced cell damage and inflammation in DED.

Dry eye disease (DED) is a relatively common condition worldwide, with a prevalence ranging from 5% to 50%, notably higher in Asian populations.^1^ DED significantly impacts individuals' visual function and life quality, thus become a considerable burden to patients and society.^2^ The Dry Eye Workshop II (DEWS II) defines DED as a multifactorial disease, with inflammation recognized as the core pathological mechanism.^3^ In our previous study, the activation of ferroptosis in corneal epithelial cells was suggested to be the main trigger for amplification of ocular surface inflammation in DED.^4^ Ferroptosis is a recently classified form of programmed cell death, closely associated with excessive oxidative stress and induction of strong inflammation.^5^^,^^6^ Ferroptosis has been suggested to be involved in the progression of numerous ocular disease.^7^^–^^9^ However, the precise processes and mechanisms underlying ferroptosis in DED remain poorly understood. It is reported that lipid peroxidation is the hallmark of ferroptosis as its direct impact on loss of cell membrane.^10^ The primary substrates of lipid peroxidation are polyunsaturated fatty acids, thus abnormal lipid metabolic pathways usually directly or indirectly regulate the lipid peroxidation and ultimate ferroptosis.^11^ Therefore, this suggests the importance of attention towards on the alterations in cellular lipid metabolism to elucidate the mechanisms underlying the induction of ferroptosis in corneal epithelial cells in DED.

Macroautophagy (hereafter autophagy) is a catabolic process that facilitates the turnover of cellular contents via the formation of autophagosomes, which subsequently fuse with lysosomes for degradation and recycling.^12^^,^^13^ Autophagy plays a pivotal role in the regulation of cellular proteins and organelles, with both excessive and impaired autophagy exerting detrimental effects on cellular physiology and influencing cell fate.^14^ Emerging evidence suggests that autophagy plays a significant role in the pathogenesis of DED. Notably, autophagy markers, including autophagy-related protein 5 (Atg5) and Lc3b-II/I, are elevated in the tears of patients with Sjögren's syndrome, and reduced levels of these markers have been associated with improved clinical signs.^15^ Enhanced autophagy has also been detected in corneal epithelial cells of DED models, but promoting autophagy is suggested to alleviate ocular surface damage caused by benzalkonium chloride.^16^ Additionally, restoring autophagy levels has been shown to relieve DED symptoms in mice following lacrimal gland excision.^17^ Thus the changes and roles of autophagy in DED may vary depending on the underlying causes. As research into autophagy progresses, a growing number of selective autophagy subtypes, like mitophagy, proteophagy, lipophagy, and nucleophagy,^18^ are being identified, highlighting the necessity for further exploration of these specific autophagy mechanism in DED.

Recent studies have highlighted the intimate correlation between ferroptosis and autophagy, particularly its selective forms, including ferritinophagy, lipophagy, clockophagy, and chaperone-mediated autophagy,^19^ in which lipophagy is recognized as a key player due to its regulatory impact on lipid peroxidation, the executor of ferroptosis.^20^ Lipophagy is a recently discovered selective autophagic pathway that specifically targets cellular lipid droplets (LDs).^21^ In this process, autophagosomes engulf LDs and transport them to lysosomes for degradation by lysosomal acid lipase, ultimately leading to the release of free fatty acids (FFAs).^22^ The accumulation of FFAs promotes the generation of PUFAs, which serve as substrates for lipid peroxides, thereby triggering ferroptosis.^23^ Excessive lipophagy has been implicated in the activation and progression of ferroptosis across various pathological conditions, such as drug resistance in head and neck cancer,^24^ heightened sensitivity to ferroptosis in hepatocytes,^25^ and renal tubular epithelial cell damage in acute kidney injury.^26^ In DED conditions, the alterations of lipophagy and its role in determining ferroptosis related cell fate of corneal epithelial cells necessitate further investigation.

Atg5, a pivotal member of the autophagy-related gene family, is central to the regulation of autophagosome formation and maturation within autophagic pathway.^27^ Emerging research implicates Atg5 as a significant regulator of lipophagy in diverse cells.^28^^,^^29^ Suppression of Atg5 expression was observed to rescue the reduction in LD abundance in hepatic stellate cells.^30^ Moreover, Atg5 knockdown has been reported to obstruct ferroptosis by inhibiting lipophagy in hepatocytes.^25^ The precise mechanisms by which Atg5 mediating lipophagy and its implications for cell homeostasis in cornel epithelial cells in DED are yet to be defined.

In this study, we demonstrated that lipophagy was induced in corneal epithelial cells within both in vitro and in vivo DED models. Atg5 was upregulated to mediate the lipophagy, altering the LD catabolism and cellular FFA contents, ultimately promoting the activation of ferroptosis in corneal epithelial cells. Silencing Atg5 was found to suppress ferroptosis and downregulate ocular surface inflammatory response in DED model mice. Collectively, our findings revealed the critical role of Atg5-mediated lipophagy in the induction of ferroptosis in corneal epithelial cells, thereby suggesting a potential therapeutic target for alleviating ferroptosis-related cell damage and downstream inflammation in DED.

## Material and Methods

### Reagents and Antibodies

Dulbecco's modified Eagle's medium/F12 medium, fetal bovine serum, recombinant human epidermal growth factor, and an insulin-transferrin-selenium supplement, penicillin/streptomycin, 0.25% trypsin-EDTA were purchased from Invitrogen/Gibco (Carlsbad, CA, USA). A Cell Counting Kit-8 (CCK- 8, no. CK04) was purchased from Dojindo Laboratories (Kumamoto, Japan). BODIPY 493/503 (D2191) and Lysotracker Red DND-99 (L7528) were purchased from Invitrogen. ATGListatin (HY-15859), Bafilomycin A1(HY-100558), Rapamycin (HY-10219) were purchased from MedChemExpress (Monmouth Junction, NJ, USA). Antibodies used in this study are listed in the Table.

**Table.** The Information of Antibodies

Category	Antibody	Company	Catalog Number	Applicants
Autophagy marker	Anti-Atg5	Cell Signaling Technology	12994	WB, CO-IP
	Anti-Atg5	ABCEPTA	AP63838	IF
	Anti-LC3B	Cell Signaling Technology	3868	WB
	Anti-Atg16L1	Abcam	ab188642	WB
	Anti- Beclin1	Cell Signaling Technology	3495	WB
LD marker	Anti-Perilipin1	Cell Signaling Technology	9349T	WB
	Anti-Perilipin2	Cell Signaling Technology	45535S	WB
	Anti-Perilipin3	Abcam	ab47638	WB, CO-IP, IF
Ferroptosis marker	Anti-Glutathione Peroxidase 4 (Gpx4)	Abcam	ab125066	WB
	Anti-Transferrin Receptor (TFRC)	Cell Signaling Technology	#46222	WB
	Anti-4-Hydroxynonenal (4-HNE)	Abcam	ab48506	WB

### Cell Culture and Treatment

The human SV40 immortalized corneal epithelial cell line (CRL-11135, HCE-2; ATCC, Manassas, VA, USA) were cultured on plates in a humidified environment with 5% carbon dioxide (CO_2_) at 37°C. The culture medium was the same as previously reported.^4^ The cells were then treated with isotonic (312 mOsM) or hyperosmotic (500 mOsM) medium for two, six, 12, and 24 hours. The hyperosmotic medium was prepared by adding 94 mM sodium chloride, following previously established protocols.^4^ The cells were treated in hyperosmotic medium for establishment of in vitro DED model. For regulating autophagy, human corneal epithelial cells (HCECs) were cultured in normal or hyperosmotic medium with or without Bafilomycin A1 (Baf1, 200 nM) or Rapamycin (Rapa, 200 nM) for 24 hours. Then, the cells were collected for LD staining and western blot assay (Perilipin1, Perilipin2, and Perilipin3).

### Lipidomic Analysis

HCECs exposed to normal or hyperosmotic medium for 24 hours were harvested and rapidly frozen in liquid nitrogen for two minutes, followed by thawing on ice for five minutes and vortex blending. This freeze-thaw cycle was repeated three times. Subsequently, the cell lysate was spun in a centrifuge at 5000 rpm at 4°C for one minute. The supernatant was homogenized using a 1 mL mixture containing methanol, methyl tert-butyl ether, and an internal standard mixture and then vortexed for 15 minutes. Water 200 µL was added and vortexed for one minute. After this, the mixture was spun in a centrifuge at 12,000 rpm at 4°C for 10 minutes. The 500 µL supernatant was extracted and concentrated. The concentrated sample was dissolved in 200 µL of mobile phase B and stored at −80°C. The dissolved sample was transferred into the sample bottle for LC-MS/MS analysis.

### RNA-Sequencing (RNA-Seq) Analysis

RNA was extracted from HCECs using the RNeasy Mini Kit (no. 74106; Qiagen, Valencia, CA, USA). RNA-seq libraries were prepared with the TruSeq Stranded mRNA Library Prep Kit (no. 20020594; Illumina, San Diego, CA, USA). Total RNA sequencing was conducted with Illumina PE150 sequencers. The sequence data were mapped to the hg19 reference genome using the STAR aligner. Transcript abundance was quantified as transcript per million, and differential expression analysis was assessed with the DESeq2 package. Genes were flagged as significantly differentially expressed if *P* < 0.01 and log2-fold change > 1. Each group included four biological replicates for RNA-seq analysis. The heatmap presented in this article was generated using “pheatmap” in *R* programming language.

### Transmission Electron Microscopy

After being treated with normal or hyperosmotic medium, HCECs were fixed with 2.5% glutaraldehyde mixed with 150 mM sodium cacodylate buffer at pH 7.4 and kept at 4°C overnight. Afterward, they underwent secondary fixation with 1% osmium tetroxide (OsO4), the were treated with uranyl acetate. The cells were dehydrated through an ethanol series and finally embedded in epoxy resin. The sections were observed under a Hitachi transmission electron microscope (HT7700; Hitachi, Tokyo, Japan).

### LD Staining

For cellular LD staining, HCECs were cultured in 24-well plates with a round cover slip placed in each well. After treatment under different experimental conditions, the cells were rinsed with PBS and fixed using 4% paraformaldehyde for 10 minutes at room temperature. Subsequently, the cells were treated with 200 µL BODIPY 493/503 (1 µg/mL) at 37°C for 15 minutes and further stained with DAPI.

For corneal LD staining, the frozen sections of mice's eyeballs were incubated with 20 µL BODIPY 493/503 (2 µg/mL) at 37°C for 15 minutes and further stained with DAPI. A confocal laser scanning microscope (LSM 980; Zeiss, Oberkochen, Germany) was used to acquire images. Fluorescence intensity was quantified with ImageJ software. Specifically, green channel images were firstly converted to “eight-bit,” and a threshold was set to identify stained regions corresponding to Bodipy-positive areas. The “Measure” function in ImageJ was used measure the area of the stained regions. The number of cells was counted manually. The area of Bodioy per cell was calculated using the formula: Area of Bodipy per cell (µm^2^) = positive area (µm^2^)/ number of cells.

### Lysosome Staining and Colocalization Analysis

For lysosome staining, HCECs were cultured in 24-well plates with a round cover slip placed in each well. After being treated with normal or hyperosmotic medium for two, six, 12, 24 hours, the cells were rinsed with PBS and further treated with 200 µL Lysotracker Red DND-99 (300 nM) at 37°C for 15 minutes before fixation. After the fixation with 4% paraformaldehyde for 10 minutes, the further staining (BODIPY 493/503 and DAPI) could be applied. Images were then obtained through a confocal laser scanning microscope. The analysis of colocalization was performed using ImageJ software. In brief, the green and red channel images were first converted to eight-bit format. The “colocalization” function in ImageJ was then used to analyze and label the colocalization area, calculate the Pearson's *R* value, and generate the corresponding figure.

### Western Blot Analysis

A Minute Total Protein Extraction Kit (no. SD001; Invent Biotechnologies, Plymouth, MN, USA) was used to extract the total cellular and corneal protein. The protein concentration was measured with a bicinchoninic acid protein assay kit (Millipore, Billerica, MA, USA). The specific way was described in our previous article.^4^ Briefly, protein samples, measured equally, were loaded onto sodium dodecyl sulfate-polyacrylamide gels for electrophoresis. After separation, the proteins were transferred onto polyvinylidene fluoride membranes. The membranes were blocked with 5% nonfat milk in Tris-buffered saline solution containing Tween 20 for two hours. Subsequently, they were incubated with primary antibodies overnight. After thorough rinsing, the membranes were incubated with secondary antibodies for one hour at room temperature. An ECL kit (no. E411; Vazyme Biotech Co., Ltd, Nanjing, China) was used to amplify the horseradish peroxidase signals, which were then visualized using a Bio-Rad Western blot detection system (Bio-Rad Laboratories, Inc., Berkeley, CA, USA). Semiquantitative analysis of grayscale images of the Western blots was performed using ImageJ software.

### RNA Interference

HCECs were transfected using Lipofectamine RNAiMAX Transfection Reagent (no. 13778150 Invitrogen), incorporating the following siRNAs: Atg5-sense, 5′-CUUGUUUCACGCUAUAUCATT-3′, antisense, 5′-UGAUAUAGCGUGAAACAAGTT-3′. A nontargeting scramble siRNA was served as a negative control. The transduction method was as previously described.^4^ In brief, 5 µL of siRNA (10 µM) and 9 µL of Lipofectamine RNAiMAX were separately added to 150 µL of Opti-MEM. Then they were mixed and incubated at room temperature for five minutes. Subsequently, the prepared mixture was added to the plates. After a 24-hour period, the medium was exchanged for either normal medium or hyperosmotic medium. After an additional 24 hours of culture, cells were collected for cell viability and lipid peroxidation assays. At six and 24 hours, cells were collected for LD staining and Western blotting assay (Atg5, Perilipin1, Perilipin2, Perilipin3, Gpx4, and Tfrc). Additionally, at two, six, and 24 hours, the FFA content was measured.

### Atg5 Overexpression

The gene human Atg5 was cloned into PDS279_pL-CMV-GFP-ccdB-puro by Bhel-Ascl. Tsingke Biotechnology (Beijing, China) was responsible for designing and constructing the Atg5-specific overexpression vector, as well as the control empty vector. The recombinant lentivirus was produced from 293T cells transfected by the recombinant lentiviral plasmid. The transduction method was as previously described.^4^ HCECs were cultured in six-well plates. When the HCECs grew for 40% to 60% confluence, the corresponding lentivirus was mixed with the medium to treat the HCECs for 36 hours. Then the cells were treated with normal medium or hyperosmotic medium for six hours for LD staining and Western blot assay (Atg5, Perilipin1, Perilipin2, and Perilipin3). After subjected to normal medium or hyperosmotic medium for six and 24 hours, the cells were collected to assess the expression of Atg5, Gpx4 and Tfrc using Western blot assay.

### Co-immunoprecipitation

The co-immunoprecipitation was performed with an immunoprecipitation Kit (HRP-Anti-Rabbit IgG Light Chain) (PK10008; Proteintech, Rosemont, IL, USA) as the manufacturer's protocol. In brief, the cell cultured in the six-well plates was digested with 0.25% trypsin-EDTA and washed with PBS for three times. After being spun in a centrifuge at 500*g* for five minutes at 4°C, the cells were collected and homogenized in 100 µL IP lysis buffer (including the protease inhibitor). Then the solution was kept on the ice for 30 minutes with gentle inversion every 10 minutes. Next, the cells were lysed by ultrasonic waves at 180 W power (sonication two seconds and off two seconds cycle) for one minute on the ice. The solution was kept on ice for 40 minutes with gentle inversion every 10 minutes and then spun in a centrifuge at 10,000*g* for 15 minutes at 4°C. The upper solution was collected, and its concentration was measured. Equal amounts of protein samples were added into the spin columns with end caps at the bottom, and the specific antibody (Atg5, 1:100; Perilipin3, 1:100) and control IgG diluted with 300 µL of incubation buffer were also added into the spin columns. Mixed samples were incubated overnight at 4°C with vertical rotation. Resuspended rProtein A/G beads slurry 50 µL was added into spin columns to precipitate immune complexes and incubated at 4°C with rotation for one to four hours. Then the end caps were taken out for flowing out and discarding the supernatant. The precipitation complex was washed with 800 µL 1 × washing buffer four times, and the solutions in collection tubes were discarded. After being spun in a centrifuge at 500*g* for 30 seconds at 4°C, 40 µL Elution buffer was added into spin tubes to elute the immunoprecipitates. Then the immunoprecipitates were analyzed by Western blot.

### Measurement of Cellular Free Fatty Acid

The content of cellular free fatty acid was measured with a free fatty acid quantitation kit (MAK044; Sigma-Aldrich Corp., St. Louis, MO, USA) according to the manufacturer's protocol. In brief, HCECs were grown in normal or hyperosmotic medium in dishes for the set time, then the equal number of cells in each group were homogenized in 200 µL of a 1% (w/v) Triton X-100 in chloroform solution. The samples were spun in a centrifuge at 13,000*g* for 10 minutes to remove insoluble material. The lower phase was collected and air dried at 50°C for 30 minutes to remove chloroform. Then the dried lipids were dissolved in 200 µL of fatty acid assay buffer by vortexing extensively for five minutes. Then 2 µL of ACS reagent was added to each sample well and incubated for 30 minutes at 37°C. Next, 50 µL of the Master Reaction Mix (comprising 44 µL fatty acid assay buffer, 2 µL fatty acid probe, 2 µL enzyme mix, and 2 µL enhancer) was added to each of the wells and mixed well using a horizontal shaker or by pipetting, and incubating the reaction for 30 minutes at 37°C in a dark environment. A microplate reader (BioTek Instruments, Winooski, VT, USA) was used to measure the absorbance at 570 nm.

### Measurement of Lipid Peroxidation

The levels of lipid peroxidation were evaluated through the application of C11-BODIPY581/591, as previously described.^4^ In brief, HCECs with transduction of Atg5-specific siRNA and scramble siRNA were grown in normal or hyperosmotic medium in six-well plates for 24 hours. The cells were then rinsed and incubated with 800 µL C11-BODIPY working solution (5 µM) in tubes at 37°C for 30 minutes. During the period of incubation, the tubes were shaken every five minutes. Then, the levels of lipid peroxidation were detected using flow cytometry (BD LSRFortessa, San Jose, CA, USA).

### Animal Model and Treatment

Female C57BL/6J mice aged six to eight weeks were purchased from Beijing Vital River Laboratory Animal Technology Co., Ltd. (Beijing, China). The DED mouse model was established using desiccation stress (DS) treatment, as described in our previous article.^4^ Specifically, C57BL/6J mice received subcutaneous injections of scopolamine hydrobromide (1.5 mg/0.3 mL; Sigma-Aldrich Corp.), administered three times daily for a period of five consecutive days. In the meantime, these mice were maintained in a controlled environment with 30% humidity or less. The matched mice in the control group were housed in an environment with 50% to 75% humidity. A phenol red thread (Jingming, Tianjin, China) was used to quantify the volume of aqueous tear secretion. The small interfering RNA modified with 2′-O-Methyl C/G/U/A and cholesterol (2′-OMe-Chol-siRNA) targeting Atg5 was synthesized by Tsingke Biotechnology (Beijing, China). The Atg5 sequence is as following, sense: 5′-CA(mU)(mG)AA(mA)A(mU)CCA(mG)AA(dT)(dT)-3′; antisense: 5′- (mU)(mU)C(mU)(mG)(mG)A(mU)A(mU)(mU)CCA(mU0(mG)(dt)(dT)-3′. The mice were subconjunctivally injected with 5 µL of 2′-OMe-Chol-siRNA targeting Atg5 or scramble siRNA 24 hours before desiccation stress treatment. Corneal damage was evaluated using sodium fluorescein staining and images were captured using a slit-lamp microscope imaging system with a green filter. Corneal staining area were quantified with ImageJ software, and the process is illustrated in Supplementary Figure S1. Briefly, the images were imported into ImageJ software, and then the color channels were split into red, green, and blue. Because of the application of a green filter during image capture, the green channel representing sodium fluorescein staining was selected for analysis. The corneal area was manually delineated using the round selection tool. The "threshold" function was then applied to identify the regions of staining, corresponding to sodium fluorescein-positive areas, with the same parameters used for all images to ensure consistency. Finally, the "measure" function was utilized to calculate the percentage of corneal staining area: Corneal Staining Area (%) = (Fluorescein sodium-positive area/Total corneal area) × 100%. All animal experiments complied with the ARVO Statement for the Use of Animals in Ophthalmic and Vision Research and were approved by the Institutional Review Board of Zhongshan Ophthalmic Center (Guangzhou, China; approval ID: 2020-138).

### Statistical Analysis

All data are presented as the mean ± standard deviation (SD). Statistical analyses were performed using GraphPad Prism (GraphPad, San Diego, CA, USA). The statistical significance of differences among multiple groups was assessed by one-way analysis of variance and a Tukey post-hoc test. A *P* value less than 0.05 was considered statistically significant.

## Results

### Lipophagy Is Activated in HCECs Under Hyperosmotic Stress

**Figure 1.**
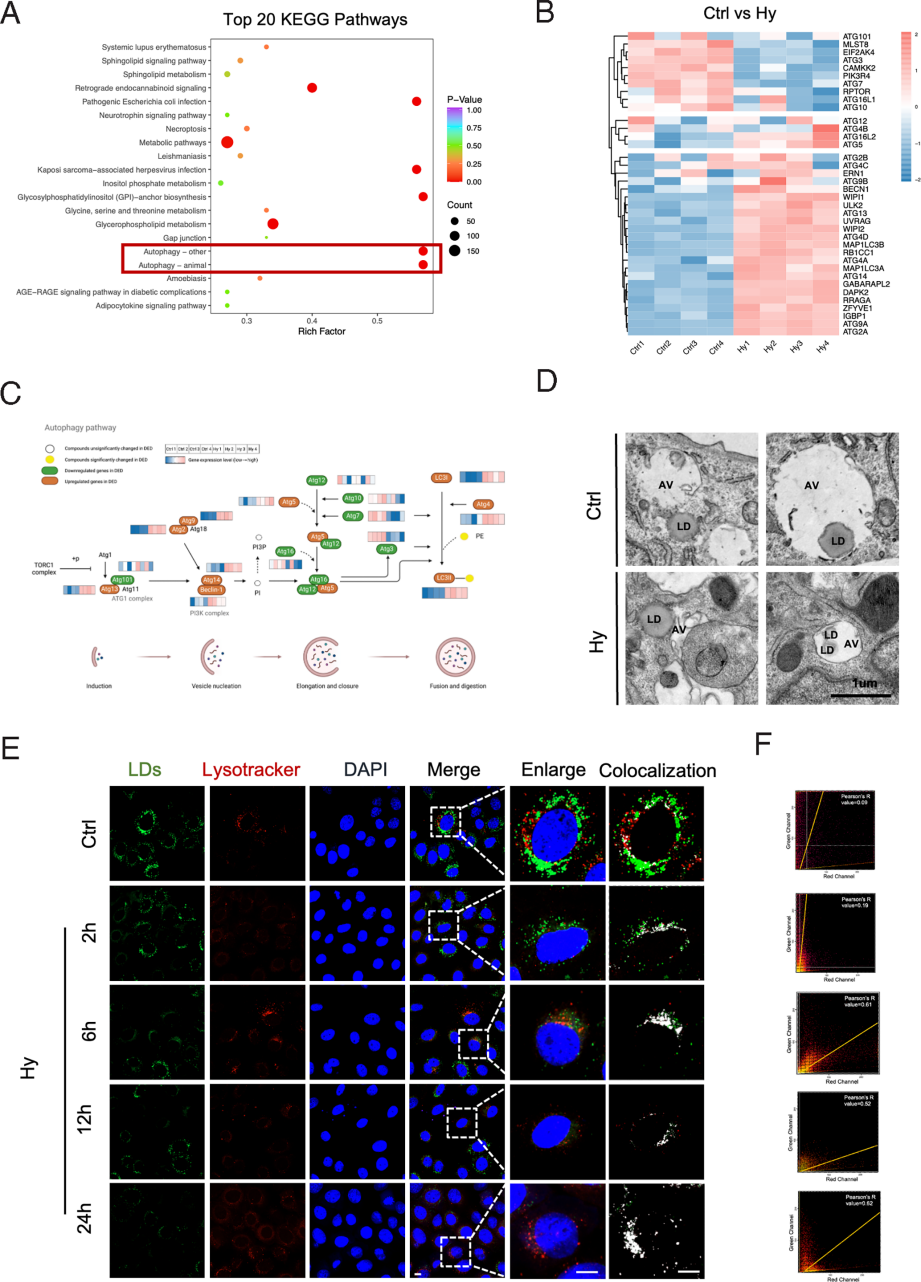
Lipophagy was activated in HCECs under hyperosmolarity. (**A**) HCECs cultured in normal medium (Ctrl) or hyperosmotic medium (Hy) for 24 hours were performed lipidomic. Top 20 Kyoto Encyclopedia of Genes and Genomes pathway enrichment analysis of differential lipids from lipidomic results. (**B**) Heatmap showing the transcriptional changes in genes in autophagy-related pathways in the Ctrl and Hy groups. (**C**) The autophagy pathway diagram presenting alterations of lipids and genes in HCECs in DED based on the results of transcriptomic and lipidomic. PE, phosphatidylethanolamine. (**D**) HCECs were cultured in normal medium or hyperosmotic medium for 24 hours. Representative ultrastructural micrographs were acquired by transmission electron microscopy. *Scale bar:* 1 µm. (**E**) HCECs were cultured with normal or hyperosmotic medium for 24 hours and then stained with Lysotracker, BODIPY 493/503 and DAPI. Boxed areas were enlarged and placed on the right side. The colocalization areas calculated by ImageJ were highlighted in *white*. (**F**) Pearson's correlation analysis was conducted to assess the colocalization in the magnified images. The graph features an orange line that represents the Pearson's *R* value. *Scale bar:* 10 µm.

As the accumulation of lipid peroxides serves as the executive factor triggering ferroptosis, we investigated and compared lipid metabolism in HCECs treated with normal or hyperosmotic medium via lipidomic detection. The lipidomic analysis revealed significant changes in lipid compositions in HCECs exposed to hyperosmolarity compared to the control group (Supplementary Fig. S2). Subsequent Kyoto Encyclopedia of Genes and Genomes pathway enrichment analysis of differentially expressed lipids between the two groups identified the obvious activation of the autophagy-other and autophagy-animal pathways in HCECs under hyperosmotic stress (Fig. 1A). To further explore the changes in transcriptional levels within autophagy pathways, RNA-seq analysis was conducted. The genes associated with autophagy-other and autophagy-animal pathways were screened out. As depicted in Figure 1B, the majority of these genes were found to be upregulated in HCECs exposed to the hyperosmotic medium. Subsequently, these genes were mapped onto the autophagy pathway along with the lipidomic results (Fig. 1C). Significant upregulated in gene expression throughout the stages of autophagy, including induction (Atg13), vesicle nucleation (Atg9, Atg2, Atg14, and Beclin-2), membrane elongation (Atg5) and the fusion of autophagosomes with lysosomes (Lc3I, Lc3II, and Atg4), collectively indicate an activated autophagy status in the in vitro DED model (Fig. 1C).

To assess the process of lipophagy, a selective autophagy specifically targeting LDs, transmission electron microscopy (TEM) was used. TEM images showed the attachment between LDs and autophagic vacuoles (AVs), with some LDs engulfed within AVs in both the Ctrl and Hy groups (Fig. 1D). Notably, a higher number of smaller LDs were observed within AVs in the Hy group. As the final step of lipophagy was achieved by lysosome degradation, we labeled lysosomes with a Lysotracker probe (Red DND-99) and LDs with a neutral lipid dye (BODIPY 493/503) to monitor their colocalization in HCECs. We found an initial overlap between lysosomes and LDs after two hours of exposure to hyperosmotic conditions, with significant colocalization evident by six hours (Figs. 1E, 1F). These findings suggest that lipophagy is activated to promote LD degradation in HCECs under hyperosmotic stress.

### Lipophagy Regulates Cellular LD Numbers

**Figure 2.**
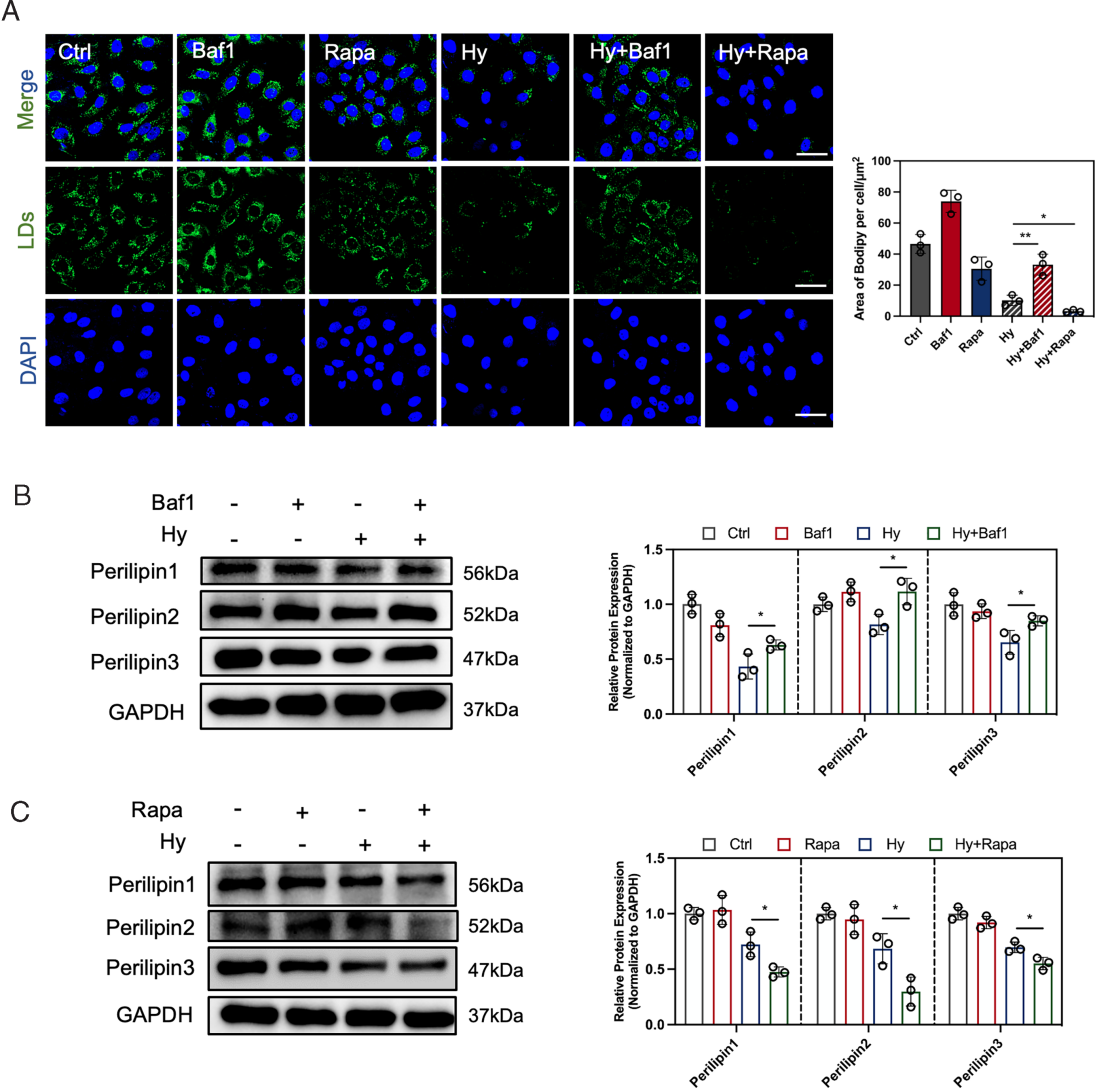
Regulation of autophagy affected the content of cellular LDs. (**A**) HCECs were cultured in normal or hyperosmotic medium with or without an autophagy inhibitor (Baf1, 200 nM) or an autophagy activator (Rapa, 200 nM) for 24 hours. Then, LDs were evaluated with the BODIPY 493/503 staining, observed by a confocal microscope (*blue*, DAPI-stained nuclei; *green*, BODIPY 493/503-stained LDs). *Scale bar:* 50 µm. The fluorescence intensity per cell was quantified by ImageJ software. (**B**) Representative immunoblots for Perilipin1, Perilipin2, and Perilipin3 in HCECs in normal or hyperosmotic medium with or without Baf1. (**C**) Representative immunoblots for Perilipin1, Perilipin2, and Perilipin3 in HCECs in normal or hyperosmotic medium with or without Rapa. GAPDH was used as the loading control. Relative protein expression levels were calculated with ImageJ and normalized to GAPDH. *N* = 3 per group. *P < 0.05; **P < 0.01.

To verify the effect of lipophagy on altering lipid metabolism, we assessed the changes of cellular LDs after regulating the autophagy. Bafilomycin A1 (Baf1), a normal autophagy inhibitor, was used to treat HCECs with or without hyperosmotic medium. The decreased cellular LD content under hyperosmotic conditions were rescued after Baf1 treatment (Fig. 2A). Because the perilipin family members are located on the membrane of LDs, their expression level can serve as indicators of LD quantity. The elevated expression of Perilipin1, Perilipin2, and Perilipin3 was observed in HCECs under hyperosmolarity with Baf1 treatment (Fig. 2B). Subsequently, rapamycin (Rapa), an autophagy inducer, was used to promote the lipophagy. As shown in Figure 2A, Rapa treatment reduced the number of LDs in HCECs cultured in normal medium and further decreased the cellular LDs under hyperosmotic conditions by promoting autophagy, as visualized by BODIPY 493/503 staining. The protein expression levels of Perilipin1, Perilipin 2, and Perilipin 3 of HCECs treated with hyperosmotic medium also decreased upon Rapa treatment (Fig. 2C). Hence, these results suggest a key role of lipophagy in maintaining cellular LD homeostasis in HCECs.

### Atg5 Is the Key Mediator of Lipophagy

**Figure 3.**
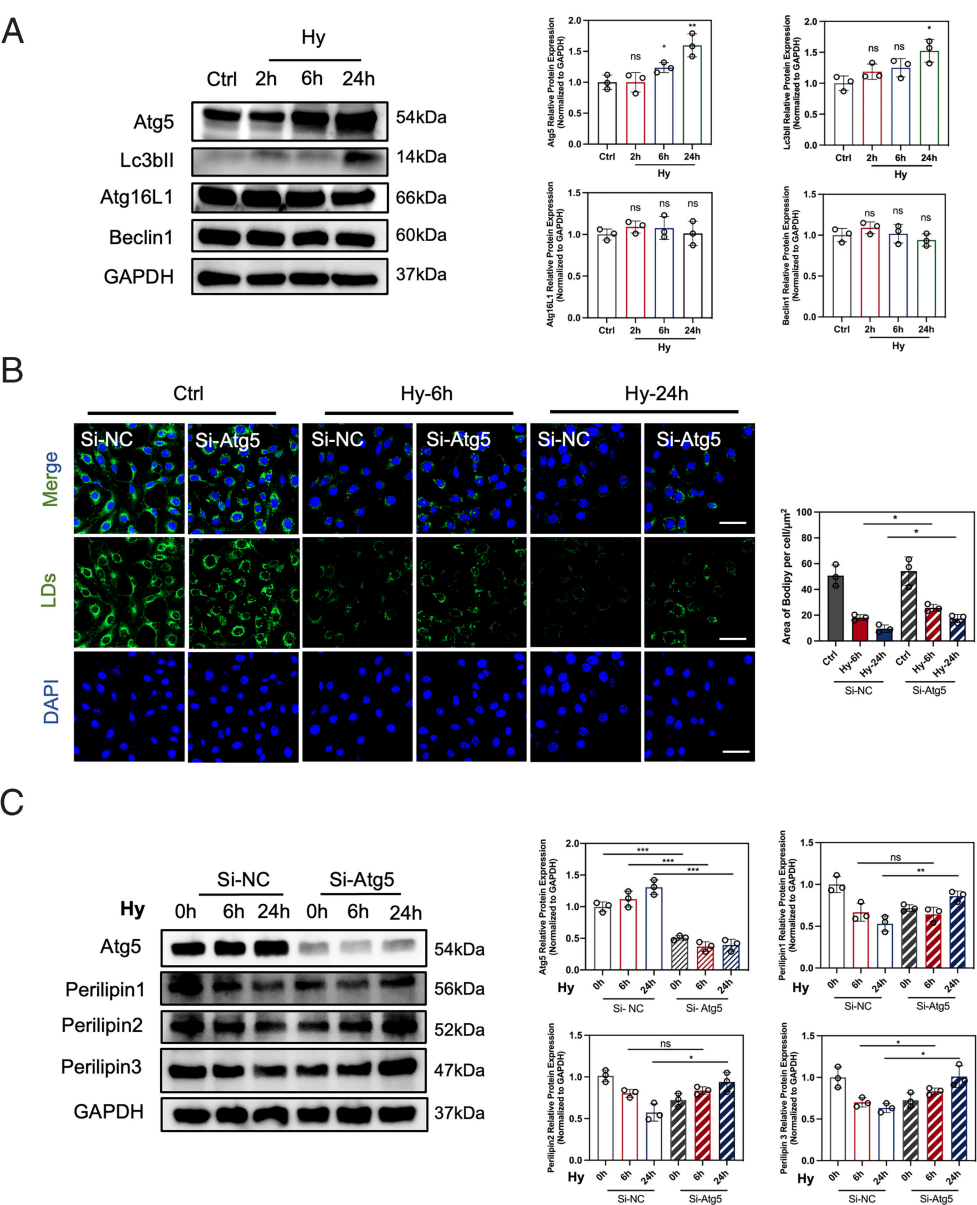
Atg5 was upregulated to mediate lipophagy under hyperosmotic stress. (**A**) Representative immunoblots for Atg5, Lc3b, Atg16L1, and Beclin1 in control or hyperosmolarity-treated HCECs for two, six, and 24 hours. *N* = 3 per group. (**B**) HCECs were exposed to the normal conditions or the hyperosmotic conditions for six and 24 hours after transfection with scramble siRNA (Si-NC) or siRNA targeting Atg5 (si-Atg5) for 24 hours. Then, LDs were evaluated with the BODIPY493/503 staining, observed by a confocal microscope (*blue*, DAPI-stained nuclei; *green*, BODIPY 493/503-stained LDs). *Scale bar:* 50 µm. The fluorescence intensity per cell was quantified by ImageJ software. (**C**) Representative immunoblots for Atg5, Perilipin1, Perilipin2 and Perilipin3. GAPDH was used as the loading control. Relative protein expression levels were calculated with ImageJ and normalized to GAPDH. *N* = 3 per group. ns > 0.05; *P < 0.05; **P < 0.01, ***P < 0.001.

To confirm the key regulators of lipophagy, we employed the western blot analysis to detect the protein expression from genes in autophagic pathways. The results depicted in Figure 3A exhibited a remarkable increase in the protein expression of Atg5 and Lc3bII in HCECs subjected to hyperosmotic medium for 24 hours. Notably, Atg5 expression was upregulated following six-hour hyperosmotic stimulation (Fig. 3A). Thus we hypothesized that Atg5 may take main responsibility for regulating lipophagy in HCECs. To clarify the role of Atg5, the small interfering RNA specifically targeting Atg5 (si-Atg5) was transfected into HCECs to inhibit Atg5 gene expression. The silencing efficiency was confirmed by western blot (Fig. 3C). Our results showed a significant increase in LDs in HCECs transfected with si-Atg5 compared to those transfected with scramble siRNA (si-NC) after both six hours and 24 hours of hyperosmotic treatment (Fig. 3B). Furthermore, we assessed the expression of LD-associated proteins Perilipin1, Perilipin2, and Perilipin3. Atg5 inhibition was associated with a marked increase in these proteins after 24 hours of hyperosmotic stress (Fig. 3C), indicating the suppression of lipophagy. These findings suggest that Atg5 is an essential mediator of lipophagy, regulating LD catabolism in HCECs under hyperosmotic stress.

### Atg5 Interacts With Perilipin3 to Induce Lipophagy

**Figure 4.**
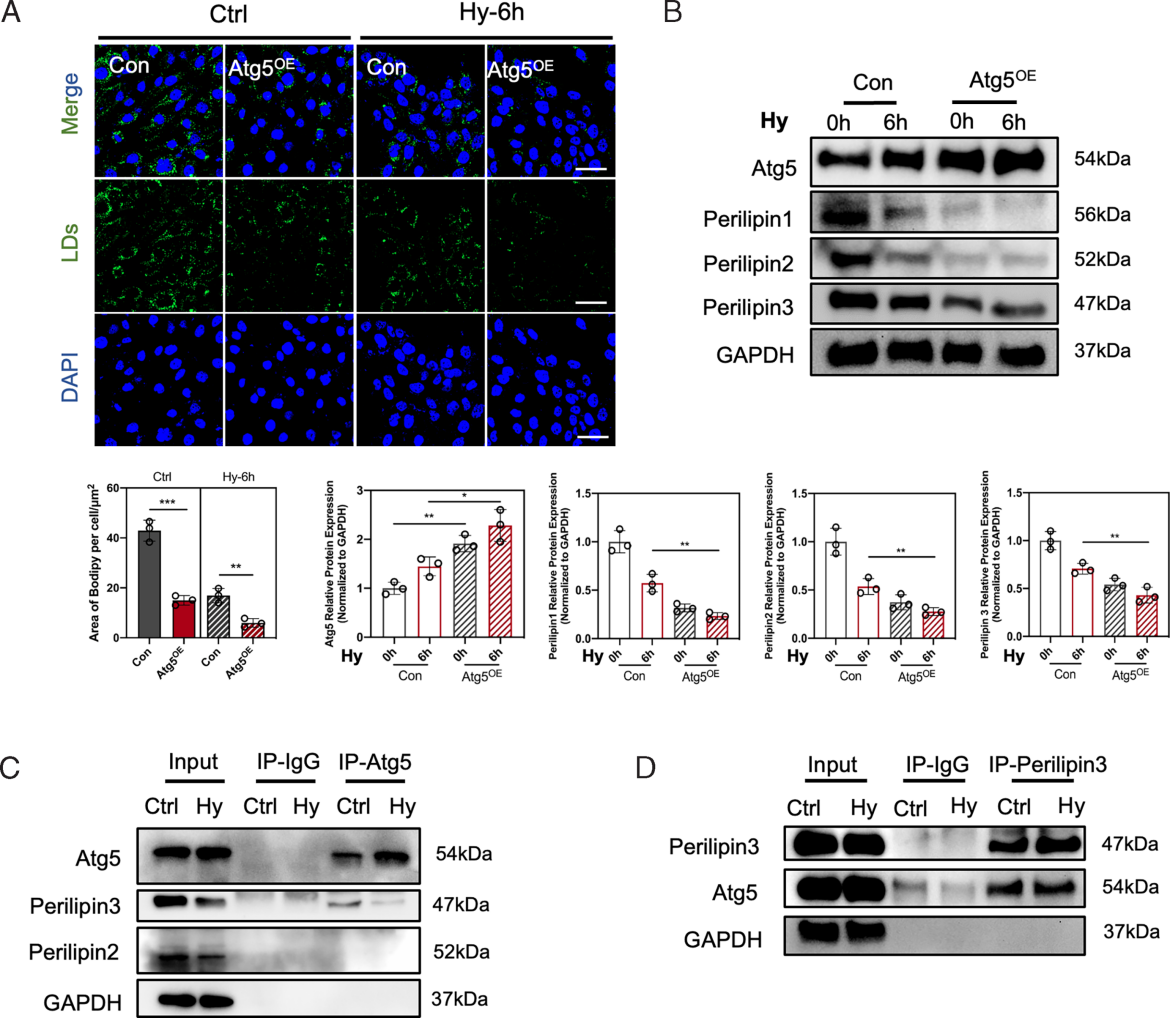
Atg5 promoted lipophagy through interacting with Perilipin3. (**A**) HCECs were exposed to normal medium or hyperosmotic medium for six hours after transduction with the Atg5-specific overexpression vector (Atg5^OE^ group) or an empty vector (Con group) for 36 hours. Then, LDs were evaluated with the BODIPY493/503 staining, observed by a confocal microscope (*blue*, DAPI-stained nuclei; *green*, BODIPY 493/503-stained LDs). *Scale bar:* 50 µm. The fluorescence intensity per cell was quantified by ImageJ software. (**B**) Representative immunoblots for Atg5, Perilipin1, Perilipin2, and Perilipin3. GAPDH was used as the loading control. Relative protein expression levels were calculated with ImageJ and normalized to GAPDH. *N* = 3 per group. (**C**) Proteins extracted from the HCECs in normal medium or hyperosmotic medium for six hours were incubated with the Atg5 antibody or IgG antibody as the probe antibody to pull down the immune complexes. Then the expression of Perilipin3 and Perilipin2 was measured by Western blot. (**D**) Proteins extracted from the HCECs in normal medium or hyperosmotic medium for six hours were incubated with the Perilipin3 antibody or IgG antibody as the probe antibody to pull down the immune complexes. Then the expression of Atg5 was measured by Western blot. *P < 0.05; **P < 0.01, ***P < 0.001.

To further validate the regulatory role of Atg5 in lipophagy process, Atg5 was overexpressed in HCECs through transduction with an Atg5-specific vector. The overexpression efficiency of Atg5 was assessed by western blot (Fig. 4B). Following the overexpression of Atg5, there was a significant reduction in the number of intracellular LDs under hyperosmotic stress, as evidenced by diminished fluorescent signal for LDs and decreased expression levels of Perilipin1, Perilipin2, and Perilipin3 (Figs. 4A, 4B). To investigate the mechanism of Atg5 mediating LD catabolism, the interaction between Atg5 and the functional proteins coating on the surface of LDs was assessed. Given the widespread expression of Perilipin2 and Perilipin3 in various cell types and their known correlation with LD catabolism, we investigated the potential interaction between those two proteins and Atg5 using co-immunoprecipitation techniques. Figure 4C demonstrates that Atg5 co-precipitated with Perilipin3 but not with Perilipin2. Correspondently, Atg5 was also detectable in the immunoprecipitation of Perilipin3 (Fig. 4D). Our results collectively suggest Atg5 is a critical mediator in lipophagy through its interaction with Perilipin3 in HCECs, highlighting a targeted pathway for regulating lipid metabolism in DED.

### Atg5-Mediated Lipophagy Regulates Ferroptosis in HCECs

**Figure 5.**
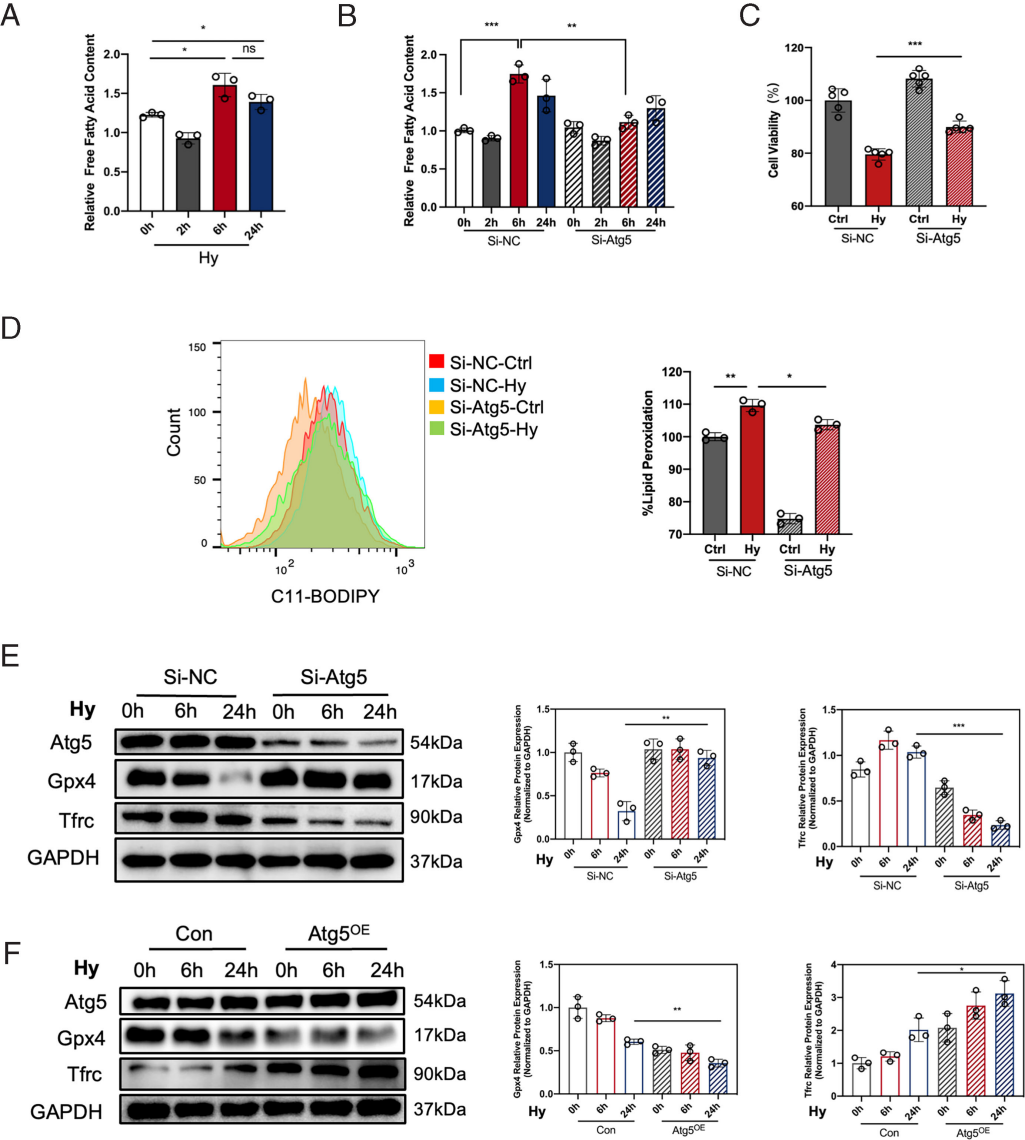
Atg5-mediated lipophagy induced ferroptosis in HCECs under hyperosmolarity. (**A**) Relative free fatty acid contents in HCECs treated with hyperosmotic medium for zero, two, six, and 24 hours were measured with a free fatty acid quantitation kit. *N* = 3 per group. (**B**) HCECs were exposed to hyperosmolarity for zero, two, six, and 24 hours after transfection with si-NC or si-Atg5 for 24 hours. Then, relative free fatty acid contents were measured with a free fatty acid quantitation kit. *N* = 3 per group. (**C**) Cell viability was measured by a CCK-8 assay. *N* = 5 per group. (**D**) Lipid peroxidation was evaluated with the BODIPY 581/591-C11 probe using flow cytometry. *N* = 3 per group. (**E**) Representative immunoblots for Atg5, Gpx4, and Tfrc in HCECs transfected with si-Atg5 or si-RNA, subjected to hyperosmotic medium for zero, six, and 24 hours. (**F**) Representative immunoblots for Atg5, Gpx4 and Tfrc in HCECs transduced with Atg5^OE^ or Con vector, subjected to hyperosmotic medium for zero, six, and 24 hours. GAPDH was used as the loading control. Relative protein expression levels were calculated with ImageJ and normalized to GAPDH. *N* = 3 per group. ns > 0.05; *P < 0.05; **P < 0.01, ***P < 0.001.

Given that enhanced lipophagy typically results in an increased release of FFAs from LDs, we subsequently evaluated the changes in cellular FFA levels under hyperosmotic stress at zero, two, six and 24 hours. Following exposure to hyperosmotic medium for six and 24 hours, a significant increase in cellular FFA content was observed (Fig. 5A), which could be abrogated by inhibiting the expression of Atg5 (Fig. 5B). The levels of cellular FFAs were suggested to be positively correlative with the occurrence of ferroptosis.^20^ Our previous research demonstrated the activation of ferroptosis in HCECs under hyperosmolarity.^4^ We found that inhibition of lipophagy through downregulating the expression of Atg5 increased cell viability and decreased the level of lipid peroxidation under hyperosmolarity (Figs. 5C, 5D). To assess the status of ferroptosis pathway in HCECs, we measured the levels of the anti-ferroptosis marker glutathione peroxidase 4 (Gpx4) and the pro-ferroptosis marker transferrin receptor (Tfrc). On exposure to hyperosmotic medium for six and 24 hours, HCECs transfected with si-Atg5 exhibited decreased expression of Tfrc and increased expression of Gpx4, in comparison to HCECs transfected with si-NC (Fig. 5E). Conversely, overexpression of Atg5 resulted in an opposite expression pattern of those ferroptosis-related markers (Fig. 5F), indicating that ferroptosis was promoted by the upregulation of Atg5. Therefore our findings demonstrate that Atg5-mediated lipophagy leads to the accumulation of cellular FFAs, thereby facilitating ferroptosis in HCECs under hyperosmotic conditions.

### Inhibition of Atg5 Expression Alleviates DED In Vivo

**Figure 6.**
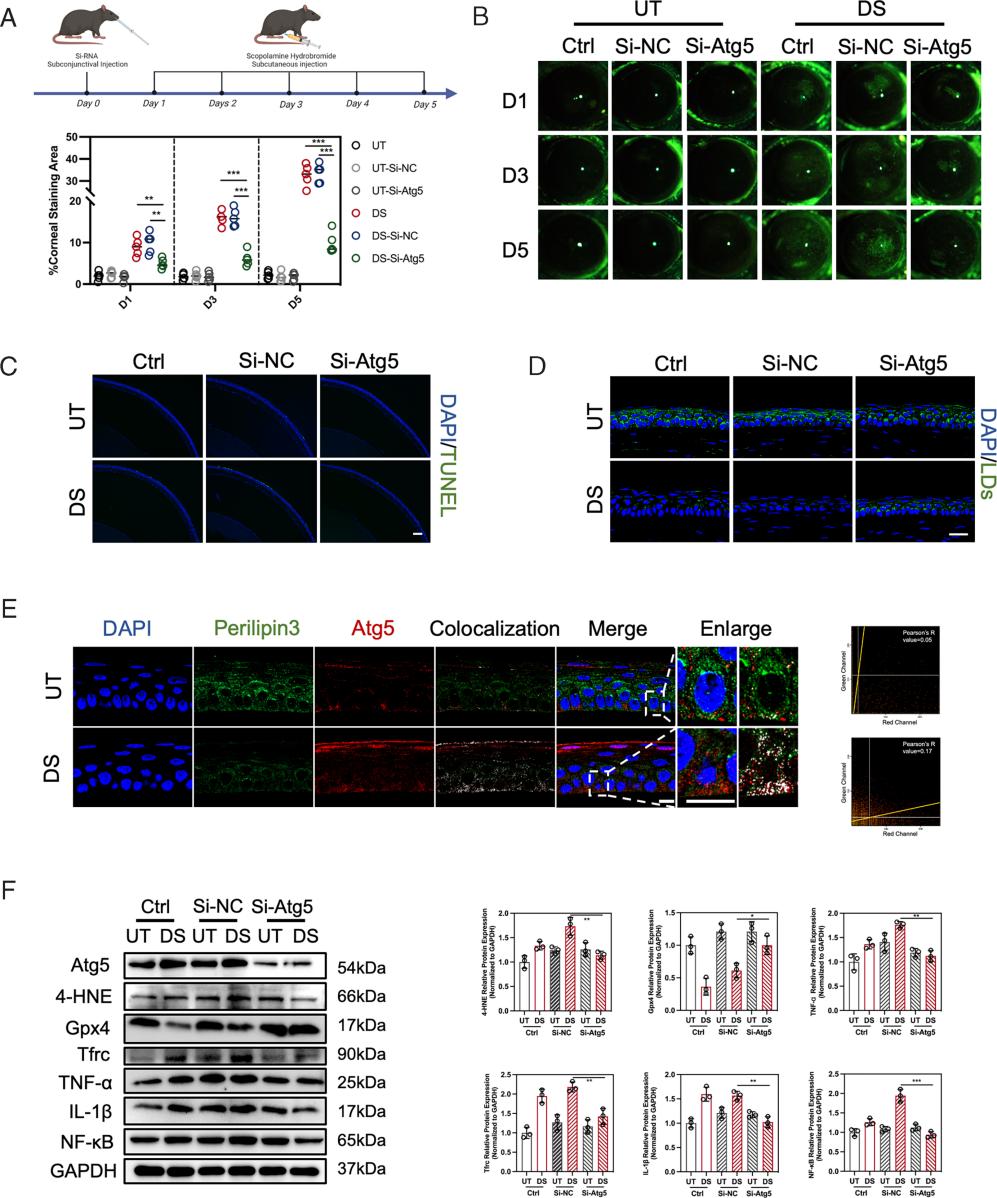
Inhibition of Atg5 alleviated corneal damage and ocular surface inflammation in the DED mouse model. (**A**) Illustration of the treatment pattern of Si-RNA and scopolamine hydrobromide on mice. 2′-OMe-Chol-siRNA was subconjunctivally injected into mice one day before the subcutaneous injection of scopolamine hydrobromide. (**B**) Representative photographs of corneal sodium fluorescein staining. The percentage of corneal staining area was calculated with ImageJ software. *N* = 5 per group. (**C**) Representative fluorescence images of TUNEL assay of the corneal epithelium (*blue*, DAPI-stained nuclei; *green*, TUNEL-positive dead cells). *Scale bar:* 100 µm. (**D**) Representative fluorescence images of corneal frozen sections (*blue*, DAPI-stained nuclei; *green*, BODIPY 493/503-stained LDs). *Scale bar:* 25 µm. (**E**) Representative fluorescence images of corneal paraffin sections (*blue*, DAPI-stained nuclei; *green*, Perilipin3-expressing cells; *red*, Atg5-expressing cells). *Boxed areas* are enlarged and placed on the right side. The colocalization areas calculated by ImageJ are highlighted in *white*. Pearson's correlation analysis was conducted to assess the colocalization in the magnified images. *Scale bar:* 10 µm. (**F**) Representative immunoblots for Atg5, 4-HNE, Gpx4, Tfrc, TNF-α, IL-1β, and NF-κB. GAPDH was used as the loading control. Relative protein expression levels were calculated with ImageJ and normalized to GAPDH. *N* = 3 per group. *P < 0.05; **P < 0.01, ***P < 0.001.

To investigate the role of Atg5 in regulating lipophagy and ferroptosis in vivo, and to explore the relationship between lipophagy and downstream inflammation, we first established a DED mouse model using DS treatment. The successful establishment of the DED model was confirmed by a significant reduction in tear volume following DS treatment (Supplementary Fig. S3). The 2′-OMe-Chol-siRNA targeting Atg5 (si-Atg5) was subconjunctivally injected into mice one day before DS treatment to specifically and efficiently inhibit the expression of Atg5 in cornea, as indicated in Figure 6A. The silencing efficiency of Atg5 in corneal epithelial cells was validated through western blot (Fig. 6F). On the first, third, and fifth day after treatment, Atg5 knockdown DED mice exhibited a significant decrease in corneal sodium fluorescein staining areas and corneal epithelial cell death, compared to the DED mice injected with scramble 2′-OMe-Chol-siRNA (si-NC) (Figs. 6B, 6C). Interestingly, the administration of si-Atg5 in DED mice resulted in an increase in LDs in corneal epithelium, indicating the inhibition of lipophagy (Fig. 6D). The enhanced colocalization of Atg5 and Perilipin3 in corneal epithelial cells in DED confirms the interaction between these two proteins. This observation aligns with our in vitro findings (Fig. 6E). We further investigate the alteration of ferroptosis in vivo. Compared to the Si-NC-DS group, the mice in the Si-Atg5-DS group exhibited decrease expression levels of 4-HNE and Tfrc, along with increased expression levels of Gpx4, suggesting that Atg5 inhibition suppressed ferroptosis in vivo (Fig. 6F). Notably, the proinflammatory cytokines TNF-α, IL-1β, and NF-κB exhibited reduced expression in DED mice after si-Atg5 interference. These results support the inhibition of lipophagy through silencing Atg5 expression, which can effectively suppress ferroptosis in corneal epithelial cells and ameliorate ocular surface inflammation in DED.

## Discussion

Our previous study demonstrated that ferroptosis activation in corneal epithelial cells promotes corneal defects and ocular surface inflammation in DED.^4^ Lipophagy is a selected autophagy process mediating cellular LD degradation, subsequently regulating the FFAs contents, ultimately determining ferroptosis-related cell fate.^31^ In this study, we revealed the activation of lipophagy in HCECs under hyperosmotic stress. Atg5 was upregulated to mediate the lipophagy process, leading to increased cellular FFAs and inducing ferroptosis in corneal epithelial cells in DED models. This lipophagy process was facilitated by the interaction between Atg5 and Perilipin3, a LD coating protein. Overall, our study highlights the significance of Atg5-mediated lipophagy in regulating ferroptosis and maintaining corneal epithelial cell homeostasis in DED.

Lipophagy is a type of selective autophagy that targets LDs for degradation, playing an essential role in maintaining normal cellular lipid metabolism and cell homeostasis.^21^ Recently, numerous studies have highlighted the significance of lipophagy in various tissues and disease conditions, like liver, heart, and nervous system diseases.^31^^–^^33^ For instance, impaired lipophagy was induced by high-glucose in microglial, leading to the accumulation of LDs and amplified the neuroinflammatory cascades in a diabetes mouse model.^34^ Activation of lipophagy in hepatocyte could significantly alleviate both steatosis and hepatitis in a nonalcoholic steatohepatitis mouse model.^35^ Although our group has reported that aberrant autophagy promoted the progression of DED,^36^ the specific subset of autophagy involved and its relationship with corneal epithelial cell fate remain poorly understood. Here, we revealed the occurrence of lipophagy in corneal epithelial cells under hyperosmotic stress, evidenced by lipidomic and RNA-seq analysis, the presence of AVs containing LDs, as well as the colocalization of lysosomes with LDs. Autophagy inhibitor Baf1 and activator Rapa were sufficient to regulate cellular LD numbers in the in vitro DED model, indicating the regulatory role of autophagy for LDs. Furthermore, inhibition of lipophagy could effectively rescue the cell viability under hyperosmolarity and reduced the corneal damage in DED mouse model. Hence, we demonstrate that activation of lipophagy induces corneal epithelial cell dysfunction and ultimate cell death in DED. However, the involvement of other selective autophagy pathways in DED still requires further exploration.

Atg5 is a member of autophagy-related gene family, which is essential for autophagosome formation as its participation in the assembly of a multimeric complex via conjugating with other autophagy-related proteins, Atg12 and Atg16L1.^37^ Interestingly, we observed an increase in Atg5 gene expression without a corresponding increase in Atg12 and Atg16L1 in HCECs under hyperosmolarity, as indicated by our RNA-seq results. It has been reported that Atg5 can play distinct roles within a complex, such as binding with bone marrow stromal antigen 2 to assemble a complex that promotes Lc3-associated processes in HeLa cells during HIV-1 infection,^38^ or binding with Tectonic β-propeller repeat containing 1 to induce autophagosome-lysosome fusion.^39^ Thus Atg5 may be independently upregulated to mediate lipophagy via interacting with Perilipin3 in HCECs under hyperosmolarity. Numerous studies have highlighted the pivotal role of Atg5 in mediating autophagy-induced lipophagy. For example, under the starvation stress, the LD abundance in renal tubular cells was higher in Atg5 knockdown mice compared to the control group.^29^ The deletion of Atg5 in hepatocyte has been demonstrated to promote the storage of neutral lipids.^40^ Likewise, our data showed that Atg5 was obviously upregulated in HCECs under hyperosmotic stress. Atg5 knockdown restored the diminished LDs, while overexpression of Atg5 resulted in further reduction of LDs, which indicates the involvement of Atg5 in regulating lipophagy in DED. However, we observed some variation in the effect of Atg5 on perilipin expression between normal and DED conditions. Specifically, lower expression levels of Perilipins in the si-Atg5 group compared to the si-NC group under normal conditions was observed, indicating that Atg5 inhibition may suppress LD formation and perilipin expression. It is reported that autophagy can contribute to LD biogenesis, LD growth, and perilipin recruitment in certain conditions, potentially by degrading membranous organelles for fatty acid supply to LDs.^41^^–^^43^ Therefore we propose that Atg5 may be involved in LD biogenesis, and its knockdown impedes both LD formation and perilipin expression in HCECs under normal conditions. Alternatively, lipolysis might be the primary pathway for LD catabolism in HCECs in normal medium. Inhibiting Atg5 could disrupt the balance between lipophagy and lipolysis, leading to compensatory enhancement of lipolysis, which promotes LD catabolism and reduces perilipin expression. Further investigation is required to clarify the specific mechanisms by which autophagy regulates LD dynamics in HCECs under both normal and DED conditions.

Studies have shown that the induction of lipophagy is mediated by the interaction between autophagic regulators and LD coat proteins.^44^ Perilipin3 was found to be combined with focal adhesion interaction protein 200KDa, a crucial protein initiating autophagy, to facilitate lipophagy in a non-alcoholic fatty liver disease mouse model.^45^ Lc3b showed an obvious colocalization with Perlipin2 in adipocyte in high-fat diet mice.^46^ Here, we demonstrate that Atg5 regulates lipophagy through interacting with Perilipin3 in corneal epithelial cells in DED models, as evidenced by CO-IP and co-immunofluorescence staining. Herein, we illustrate a novel lipophagy pathway mediated by the interaction between Atg5 and Perilipin3 in corneal epithelial cells, proving a promising foundation for further research into lipophagy in DED.

Emerging evidence has suggested that ferroptosis is a form of cell death closely associated with autophagy.^47^ Studies have indicated that lipophagy is intimately involved in regulating ferroptosis as its impact on LD catabolism and the subsequent release of FFAs. The abundance of FFAs determines the extent of ferroptosis by affecting levels of lipid peroxidation, which is the executor of ferroptosis.^48^^–^^50^ Previous research has shown that enhanced lipophagy could increase the production of FFAs and subsequent lipid peroxidation, ultimately activates ferroptosis in hepatocytes.^25^ Consistent with these findings, our study observed a similar condition, wherein inhibition of lipophagy resulted in decreased lipid peroxidation levels in HCECs under hyperosmotic stress. Interestingly, we found that inhibition of Atg5 had no significant effect on cellular FFA content but reduced lipid peroxidation under normal conditions. We supposed that Atg5 may influence lipid peroxidation through other mechanisms, such as inducing the autophagic degradation of ferritin^51^ or mediating generation of reactive oxygen species to promote ferroptosis.^52^ Therefore Atg5 might be critical for regulating FFA content and lipid peroxidation in a context-dependent manner. Furthermore, our results showed that the suppression of lipophagy through Atg5 knockdown resulted in the downregulation of ferroptosis signals in corneal epithelial cells in both in vitro and in vivo DED models. Moreover, the inflammatory response on the ocular surface, identified as a downstream effect of ferroptosis in DED,^4^ was significantly suppressed in Atg5 knockdown DED mice. These findings indicate a potential protective mechanism against ferroptosis in corneal epithelial cells and highlight lipophagy as a promising therapeutic target for mitigating inflammation in DED.

## Conclusions

In conclusion, our study elucidates that upregulation of Atg5 mediates lipophagy, resulting in the accumulation of FFAs, activation of ferroptosis, and subsequent promotion of downstream inflammation in corneal epithelial cells in DED. Thus targeting Atg5 or modulating lipophagy may present a promising therapeutic strategy for protecting corneal epithelial cells and alleviating ocular surface inflammation in DED.

## Supplementary Material

Supplement 1
