# Supplementary material for: Atg5-Mediated Lipophagy Induces Ferroptosis in Corneal Epithelial Cells in Dry Eye Disease
Source: Invest Ophthalmol Vis Sci. 2024 Dec 5;65(14):12. doi: 10.1167/iovs.65.14.12 (PMC11622160; doi:10.1167/iovs.65.14.12)
Supplement: Supplement 1 [file iovs-65-14-12_s001.pdf]

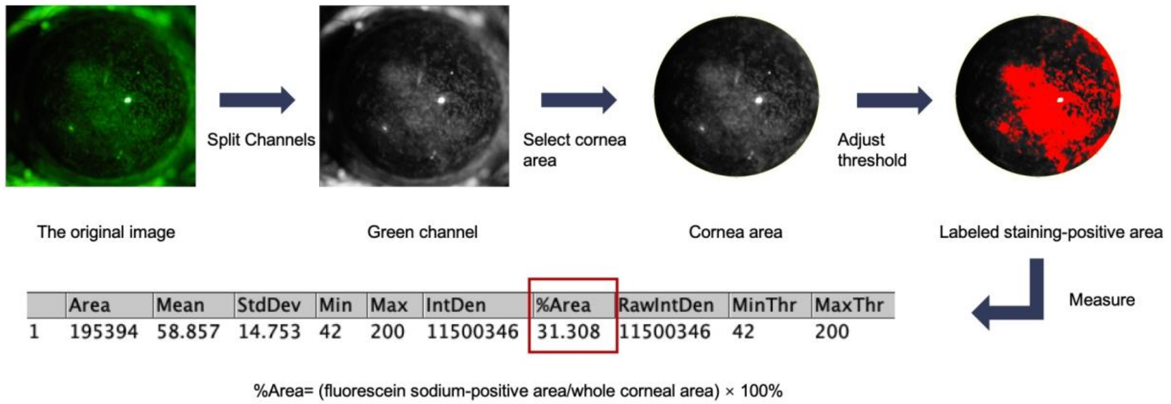

	Area	Mean	StdDev	Min	Max	IntDen	%Area	RawIntDen	MinThr	MaxThr
1	195394	58.857	14.753	42	200	11500346	31.308	11500346	42	200

%Area= (fluorescein sodium-positive area/whole corneal area) × 100%

Supplementary Figure 1. Illustration of quantification analysis of corneal staining area. Images were imported into ImageJ software, and then the color channels were split into red, green, and blue. The green channel representing sodium fluorescein staining was selected for analysis. The corneal area was manually delineated using the round selection tool. The "Threshold" function was then applied to identify the regions of staining, corresponding to sodium fluorescein-positive areas, with the same parameters used for all images to ensure consistency. Finally, the "Measure" function was utilized to calculate the percentage of corneal staining area (%Area), which representing (fluorescein sodium positive area/total corneal area) × 100%

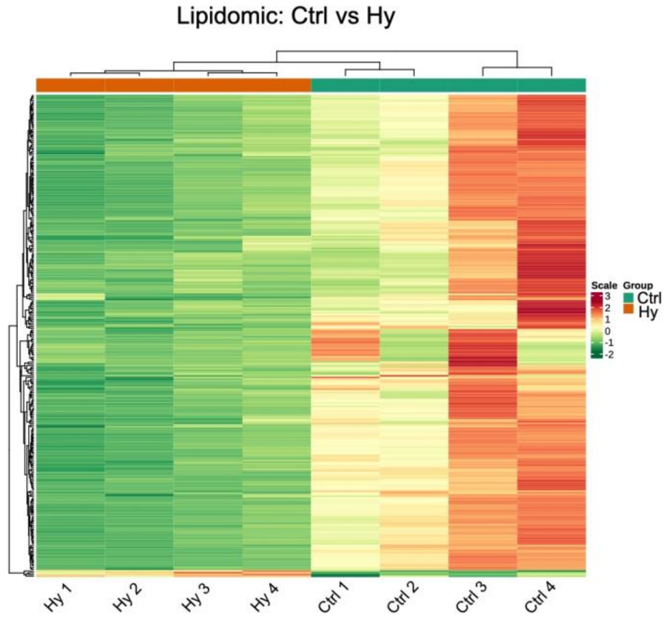

Supplementary Figure 2. Heatmap of Lipidomic. Heatmap showing the alterations of lipid composition in HCECs cultured in a normal medium (Ctrl) relative to those cultured in a hyperosmotic medium (Hy) for 24 hours (four samples per group).

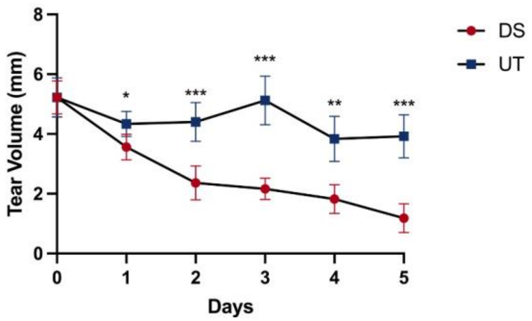

Supplementary Figure 3. Tear volume detection of wild-type mice in the DS group and UT group for 0, 1, 2, 3, 4, 5 days. The volume of aqueous tear secretion was quantified using a phenol red thread. The comparison was employed between two groups. *$P < 0.05$; **$P < 0.01$, ***$P < 0.001$.
